# Quality of Life Following Cochlear Implantation in Patients With Menière's Disease

**DOI:** 10.3389/fneur.2021.670137

**Published:** 2021-06-17

**Authors:** Isabel Sanchez-Cuadrado, Miryam Calvino, Jose Manuel Morales-Puebla, Javier Gavilán, Teresa Mato, Julio Peñarrocha, Maria Pilar Prim, Luis Lassaletta

**Affiliations:** ^1^Department of Otolaryngology, La Paz University Hospital, Madrid, Spain; ^2^IdiPAZ Research Institute, Madrid, Spain; ^3^Biomedical Research Networking Centre on Rare Diseases (CIBERER-U761), Institute of Health Carlos III, Madrid, Spain

**Keywords:** Meniere's disease, cochlear implant, quality of life, hearing loss, labyrinthectomy, vestibular neurectomy, quality of sound, matched-control evaluation

## Abstract

**Background:** Menière's disease (MD) is a disorder characterized by auditory and vestibular dysfunction that significantly deteriorates patients' quality of life (QoL). In addition to the management of vestibular symptoms, some patients with bilateral hearing loss meet criteria for cochlear implantation (CI).

**Objectives:** (1) To assess hearing results and QoL outcomes following CI in patients with MD. (2) To compare these results to a matched control group of patients who had undergone CI. (3) To analyse differences in MD patients who have undergone simultaneous or sequential labyrinthectomy or previous neurectomy.

**Methods:** A retrospective analysis of a study group of 18 implanted patients with MD and a matched control group of 18 implanted patients without MD, who had CI at a tertiary referral center. Hearing and speech understanding were assessed via pure-tone audiometry (PTA) and disyllabic perception tests in quiet. QoL was assessed *via* the Nijmegen Cochlear Implant Questionnaire (NCIQ), the Glasgow Benefit Inventory (GBI), the Speech, Spatial and Qualities of Hearing Scale (SSQ_12_), and the Hearing Implant Sound Quality Index (HISQUI_19_). The impact of MD ablative surgeries was analyzed in the study group (MD group).

**Results:** Mean pre-operative PTA thresholds were significantly lower in the MD group (103 vs. 121 dB). A significant improvement in hearing outcomes was observed following CI in both groups (*p* < 0.001), with a maximum Speech Discrimination Score of 64 and 65% disyllables at 65 dB for the MD and control group, respectively. Subjective outcomes, as measured by the NCIQ, GBI, SSQ_12_, and HISQUI_19_ did not significantly differ between groups. In the MD group, despite achieving similar hearing results, QoL outcomes were worse in patients who underwent simultaneous CI and labyrinthectomy compared to the rest of the MD group. Post-operative NCIQ results were significantly better in patients who had undergone a previous retrosigmoid neurectomy when compared to those who had undergone only CI surgery in the subdomains “basic sound perception” (*p* = 0.038), “speech” (*p* = 0.005), “activity” (*p* = 0.038), and “social interactions” (*p* = 0.038).

**Conclusion:** Patients with MD and severe hearing loss obtain hearing results and QoL benefits similar to other CI candidates. Delayed CI after labyrinthectomy or vestibular neurectomy can be performed with similar or better results, respectively, to those of other cochlear implanted patients. Patients who undergo simultaneous CI and labyrinthectomy may achieve similar hearing results but careful pre-operative counseling is needed.

## Introduction

Menière's disease (MD) is a disorder of the inner ear that causes auditory and vestibular symptoms. Different scientific societies consider that the diagnosis of definite MD relies on clinical criteria, which include: (i) two or more spontaneous episodes of vertigo (each lasting between 20 min and 12 h), (ii) with audiometrically documented low-to-medium frequency sensorineural hearing loss in one ear, defining the affected ear on at least one occasion before, during, or after one of the episodes of vertigo, (iii) fluctuating aural symptoms (hearing, tinnitus, and/or fullness) in the affected ear, and (iv) not better accounted for by another vestibular diagnosis (1).

Most patients will suffer different degrees of permanent hearing loss, and for 15-38% of patients with unilateral MD the hearing loss will progress to severe sensorineural hearing loss (2). The management of this condition aims to minimize vestibular symptoms while preserving hearing as much as possible. The majority of patients are controlled by lifestyle modifications and conservative medical treatments (Betahistine, diuretics, steroids, etc.). If unsuccessful, intratympanic injections of gentamicin or vestibular neurectomy can be proposed, with both procedures entailing the risk of hearing loss. Surgical labyrinthectomy is the most efficient treatment for vertigo attacks but this surgical procedure forfeits residual hearing (3, 4).

Advanced stages of MD are related to a decrease of subjective well-being and quality of life (QoL) due to vertigo, anxiety, limitation of life, and deafness, as well as communication problems that contribute to patients' isolation (5). The use of cochlear implantation (CI) in MD patients with bilateral severe to profound hearing loss is well-accepted, and the majority of studies show a significant improvement in post-implantation hearing and communication (6, 7). In specific cases, either simultaneous or sequential labyrinthectomy can be performed in addition to CI in order to treat both the hearing loss and the vertigo attacks (8, 9).

However, the impact of CI on the QoL of MD patients is still a controversial issue. Although an improvement of speech perception with the cochlear implant should improve social interactions and thus QoL, the few published papers about CI in MD include very small cohorts and lack specific QoL questionnaires for patients with hearing loss, which leads to uncertain results.

The purpose of this study was to review the audiological and QoL outcomes in MD patients who underwent CI, including general and specific questionnaires. A comparison of the results to a matched control group of postlingually implanted adults with similar characteristics was also performed. In addition, patients requiring ablative surgeries such as a labyrinthectomy (previous or simultaneously) or neurectomy were studied separately.

## Methods

### Study Design

A retrospective study was conducted at the Department of Otorhinolaryngology, La Paz University Hospital, Madrid, Spain. The study procedures were approved by the university hospital's ethics committee (PI-3938). The patient database for adult subjects (≥18 years) who had undergone CI between 1995 and 2019 was reviewed. Cochlear implant users who were diagnosed with MD according to the classification of Lopez-Escamez et al. (1) were selected and, from that selection, only patients with the diagnosis of “definitive Menière's disease” were included in the study for review of their medical records.

All patients underwent computed tomography (CT) and magnetic resonance imaging (MRI) to confirm cochlear patency and an intact auditory pathway prior to being considered for CI candidacy.

Assessment consisted of QoL questionnaires, and an audiological evaluation performed at last visit before surgery and at least 1 year after first fitting of the cochlear implant.

### Cochlear Implant Users

The MD group consisted of 18 patients who fulfilled the above mentioned inclusion criteria. A control group of 18 postlingually implanted users with hearing loss due to other etiologies (not related to MD) was also selected from university hospital's database of cochlear implantees by the senior otologist (L.L.), who remained blinded to audiometric and QoL data. The control group was matched to the MD group for age at implantation, gender, and type of implant. Members of both groups were unilaterally implanted.

Medical records were reviewed for demographic information, as well as for pre- and post-operative audiometric data. Patient characteristics of both groups are displayed in Table 1. In the MD group, all patients presented stage 4 MD (the worst stage and is defined by a PTA > 70 dB) in the implanted ear according to the classification scheme of the American Academy of Otolaryngology—Head and Neck Surgery (10). Eleven of these cases had bilateral MD and the other seven had unilateral MD and profound hearing loss due to other aethiologies on the contralateral ear. No comorbidities such as migraine, autoimmune disorders, or genetic factors were observed in the bilateral MD patients.

**Table 1:** Patients' characteristics.

		**MD group**	**Control group**	**p**
N		18	18	–
Age at implantation (years)^†^		59.7 ± 9.9	59.9 ± 9.9	0.946
Gender^‡^	Male	9 (50%)	9 (50%)	–
	Female	9 (50%)	9 (50%)	–
Implant^‡^	Nucleus 22M	1 (5.5%)	1 (5.5%)	–
	Nucleus 512	1 (5.5%)	1 (5.5%)	–
	Combi 40+	1 (5.5%)	1 (5.5%)	–
	Pulsar	3 (17%)	3 (17%)	–
	Sonata	8 (44.5%)	8 (17%)	–
	Concerto	4 (22%)	4 (17%)	–
Ablative surgery for MD^‡^	Simultaneous CI and labyrinthectomy	2 (11%)		
	Sequential CI and labyrinthectomy	2 (11%)		
	Previous retrosigmoid vestibular neurectomy	3 (16%)		
Hearing aid in the ear to be implanted^‡^		11 (61%)	15 (83%)	0.589 (Chi2)
Pre-op values in the ear to be implanted^†^	PTA4 (dB)	102.9 ± 27.2	121.6 ± 15.4	0.031*
	SDS (% disyllables)	22.2 ± 32.6	13.6 ± 20.4	0.606

† *Data are shown as mean ± standard deviation.*

‡ *Data are shown as absolute numbers and relative frequencies (%).*

Pre-op, preoperatively; PTA4, pure tone threshold average of 500, 1,000, 2,000, and 4,000 Hz; SDS, speech discrimination score. When no response was detected, 140 dB value was used. Control patients without MD were implanted within ± 2 years of a correspondingly matched patient with MD. *p ≤ 0.05.

In the present study, the majority of patients had no vertiginous episodes at the time of CI due to a remission of vestibular symptoms that is frequent in stage 4 of MD (8) or to previous ablative surgery. Retrosigmoid vestibular neurectomy had been performed in three cases (16%) on the ipsilateral implanted ear long before the CI and two patients (11%) have undergone sequential labyrinthectomy and CI. None of the patients with previous intratympanic gentamicin injection fulfilled the criteria for CI. Only two patients (11%) had frequent vertigo episodes and drop attacks which were resolved with simultaneous CI and labyrinthectomy, and another patient (5%) reported aural fluctuations without vertigo after an initial period of typical MD, that disappeared after CI. In the post-operative period, only patients with synchronic labyrinthectomy presented chronic imbalance and dizziness. MD patients didn't present more vestibular damage than other cochlear implant patients.

Two patients of the MD group required reimplantation: one of the patients for technology upgrade and the other due to electrode extrusion from the cochlea. No cochlea ossification was observed in the revision surgeries; reimplantation were uneventful.

### Outcome Measurements

#### Audiometric and Speech Perception Testing

Audiometric evaluation was performed in an audiometric booth with double-wall and sound isolation. The two-channel Amplaid® audiometer (Amplifon, Milan, Italy) was used for testing. If a patient had better hearing in the non-implanted ear, this ear was masked during the evaluation to reduce the binaural benefit of the non-tested ear.

All subjects underwent the following tests: preoperatively, pure tone thresholds were measured under headphones at 125, 250, 500, 1,000, 2,000, 4,000, and 8,000 Hz, as well as the maximum Speech Discrimination Score (SDS, disyllabic words in silence). Postoperatively, warble tone measurements were evaluated in a free field condition at 250, 500, 1,000, 2,000, 4,000, and 6,000 Hz with the cochlear implant. The position of the patient was 1 m away from the loudspeakers at 0° azimuth, and patient was directly facing the speakers at all times during testing. Post-operative speech perception was assessed *via* a verbal perception test of disyllabic words in same condition of free field in quiet. A recorded standard Spanish-language speech test was used (11). The lists were administered in random order at 65 dB SPL. Subjects were seated 1 m away from the loudspeakers at 0° azimuth. Subjects were assessed at least 12 months after first fitting of their audio processor.

For purposes of the statistical analyses, we considered the mean PTA thresholds at 500, 1,000, 2,000, and 4,000 Hz (PTA4) (12).

#### Quality of Life

Subjective benefit in terms of QoL was assessed with the Spanish versions of three different self-reported questionnaires: the Nijmegen Cochlear Implant Questionnaire (NCIQ), Glasgow Benefit Inventory (GBI), and the Speech, Spatial and Qualities of Hearing Scale (SSQ_12_). The HISQUI_19_ was employed to verify the subjective quality of sound. All the tests were completed at least 12 months after first fitting (NCIQ was also filled in before surgery).

The validated Spanish version of the NCIQ was used to quantify health-related QoL in cochlear implant users (13). This questionnaire provides a measure of benefit that can be used to compare the status of the individual before and after surgery. The NCIQ has six subdomains: basic sound perception, advanced sound perception, speech production, self-esteem, activity, and social interactions. The answers to the questionnaire are provided on a five-point Likert scale, with items' scores ranging from 0 (very poor) to 100 (optimal). Scores for the subdomains were computed by adding together the 10-item scores of each subdomain and dividing by the number of completed items.

The GBI is a validated QoL questionnaire developed to assess the outcome of otorhinolaryngology interventions (14, 15). It is comprised of 18 questions and generates a scale from −100 (maximal detriment) to 0 (no change) to +100 (maximal benefit). These 18 questions can be divided into three subscales: a general sub-scale (12 questions), a social support subscale (3 questions), and a physical health subscale (3 questions), and the total score (Overall Benefit) is calculated by adding the scores of all questions. Therefore, the questionnaire assesses the patient's perception at the overall success of surgery, and of the influence of CI on their phychological, social, and physical functioning.

The SSQ_12_ is a 12-item questionnaire that quantifies the severity of hearing disability. Individual items are answered on a 10-point Likert scale: the higher the score, the less disability experienced. The total SSQ_12_ score (max 10, min 0) is the average of item scores (16).

The validated Spanish language version of the HISQUI_19_ was used in this study (17). The HISQUI_19_ is a 19-item questionnaire that measures quality of sound in everyday communication situations (e.g., listening to unfamiliar speakers, understanding speech on the phone, radio or TV, etc.). Each item is answered according to frequency on a seven-point scale, the endpoints of which are “always” (seven points) and “never” (one point). The total HISQUI_19_ score is the sum of the individual item scores. Total scores are assigned a qualitative level of quality of sound: 110–133 is “very good”; 90–109 is “good,” 60–89 is “moderate,” 30–59 is “poor,” and <29 is “very poor.”

### Data Analysis

Demographic characteristics and outcome measures are shown as absolute (*n*) and relative frequencies (%) and, if appropriate, as mean plus standard deviation (± SD) and range.

The Mann-Whitney *U*-test and unpaired *t*-test (when the data are normally distributed; normal distribution was assessed by the Kolmogorov-Smirnov test and Q-Q plots) were used to examine the difference between both groups' objective and subjective measures, and to explore if any difference was found in terms of type of surgery in the MD group. Use of hearing aid in the ear to be implanted was compared between the MD group and control group with the chi-squared test.

Correlation analysis using Pearson's coefficient or Kendall's tau (normally or non-normally distributed data, respectively) was performed to evaluate the relationship between the patients' scores on the questionnaires, audiometric data, speech perception test results, and age at implantation.

Missing data and the response option “not applicable” were treated as missing values. A level of *p* ≤ 0.05 (two-tailed) was considered significant. Statistical analyses were processed in the SPSS software package v24.0 (IBM Corp., Armonk, NY, USA).

## Results

### Audiometric and Speech Perception Testing

The MD group had significantly better PTA4 before surgery than the control group, although no significant difference was found in terms of pre-operative speech discrimination. For both groups, there was a significant improvement in PTA4 from pre-operative testing to post-operative testing (*p* < 0.001). All patients' post-CI performances improved significantly in comparison to their pre-CI speech perception capabilities (Table 2). All patients from both groups were daily users of the CI.

**Table 2:** Audiometric data.

	**Pre-op**		**Post-op**	
	**PTA4 (dB HL)**	**Disyllables in**	**PTA4 (dB HL)**	**Disyllables in**
		**quiet (%)**		**quiet (%)**
MD group	103 ± 27	22 ± 33	38 ± 7	64 ± 25
Control group	122 ± 15	13 ± 20	37 ± 6	65 ± 18
*p*-value	0.031*	0.606	0.606	0.901

Results are shown as mean ± standard deviation. Pre-op, preoperatively; Post-op, postoperatively; PTA4, pure tone threshold average of 500, 1,000, 2,000, and 4,000 Hz; if no response could be elicited at the PTA, 140 dB value was used. *indicates significant difference, p ≤ 0.05.

### Quality of Life

All patients except one patient in the MD group, who refused to participate in the QoL study, answered the questionnaires.

#### NCIQ

The NCIQ evaluation showed a significant improvement in all subdomains after surgery for both groups (all *p* < 0.05). No significant intergroup differences between mean scores for any of the specified subdomains were found pre- or postoperatively (Table 3). The greatest benefit was observed in basic sound perception: scores improved 50 points in the MD group and 48 points in the control group.

**Table 3:** NCIQ scores.

	**Basic sound perception**		**Advanced sound perception**		**Speech production**		**Self-esteem**		**Activity**		**Social interaction**	
	**Pre**	**Post**	**Pre**	**Post**	**Pre**	**Post**	**Pre**	**Post**	**Pre**	**Post**	**Pre**	**Post**
MD group	24 ± 25	74 ± 18	62 ± 23	77 ± 17	23 ± 28	61 ± 22	43 ± 16	65 ± 13	36 ± 26	67 ± 29	35 ± 23	59 ± 18
Control group	27 ± 19	75 ± 24	59 ± 20	78 ± 20	31 ± 21	56 ± 24	47 ± 19	68 ± 23	43 ± 26	76 ± 25	37 ± 21	67 ± 21
*p*-value	0.463	0.613	0.757	0.613	0.163	0.546	0.660	0.546	0.369	0.287	0.732	0.134

*Results are shown as means ± standard deviation of pre- and post-operative assessments*.

#### GBI

The mean GBI scores of both groups are shown in Table 4. Even though no significant difference was observed between the two groups, the minimum value of the range was negative in all GBI subscales for the MD group. Only two patients reported negative GBI scores. These two patients underwent CI and labyrinthectomy simultaneously.

**Table 4:** GBI scores by group.

**GBI**	**MD group**	**Control group**	***p*-value**
Overall benefit	27 (−31 to 67)	29 (3 to 69)	0.807
General health	38 (−50 to 88)	40 (0 to 83)	0.732
Social benefit	12 (−17 to 50)	13 (0 to 67)	0.987
Physical health	0 (−17 to 50)	5 (0 to 50)	0.303

*Results are shown as mean (range)*.

#### SSQ_12_

The total SSQ_12_ score was 4.0 ± 1.5 in the MD group and 5.0 ± 2.0 in the control group. No significant difference was noted between the two groups, although the control group rated the degree of self-perceived hearing disability slightly higher than the MD group.

#### HISQUI_19_

Regarding the quality of sound, the HISQUI_19_ questionnaire showed no significant difference between the MD group (79 ± 26) and the control group (70 ± 24). Both groups rated the quality of sound as “moderate.”

### Relations Between Variables

The impact of variables such as age, sex, previous use of a hearing aid in the implanted ear, type of implant, and post-operative audiometric outcomes on the QoL were analyzed. Patients in the MD group who had previous hearing aid use performed better than those with no previous use of a hearing aid in the pre-operative subdomain “advanced sound perception” of the NCIQ (*p* = 0.027). Patients in the control group with previous hearing aid use performed significantly better than non-users on the HISQUI_19_ (*p* = 0.027). The hearing aid users referred to their subjective quality of sound with the CI as “moderate” (mean = 85 points) compared to the “poor” subjective quality of sound reported by the non-users (mean = 50 points). No other significant associations were found.

In the MD group, no significant difference was found between patients who underwent sequential or simultaneous labyrinthectomy with CI and those who underwent only CI in terms of auditory or QoL results. Nevertheless, despite no significant difference, when observing the results of the two simultaneous labyrinthectomy patients, a negative impact in the GBI, a post-operative decrease in some subdomains of the NCIQ and a poor benefit with the cochlear implant in the HISQUI_19_ was observed. The three patients who had undergone a previous neurectomy performed significantly better than patients who underwent only CI in the subdomains “basic sound perception” (*p* = 0.038), “speech” (*p* = 0.005), “activity” (*p* = 0.038), and “social interactions” (*p* = 0.038) of the NCIQ. No other significant differences were observed within the MD group.

## Discussion

### General Results

CI is gaining acceptance in the population who has MD when hearing improvement cannot be achieved with hearing aids. This retrospective study demonstrates that patients with MD can undergo CI surgery with similar expectations to those of other adults with postlingual severe to profound hearing loss, because significant improvement can be observed in both audiological and QoL measures in the great majority of cases.

### Difference in Pre-operative Hearing and in Hearing Aid Use Between MD and Control Groups

Hearing fluctuation is common in patients with MD, who frequently report reduced dynamic range and speech perception, which leads to difficulties in hearing aid fitting (18). In this study, the use of hearing aids in the ear to be implanted was less common in the group of patients with MD, and only 61% used a hearing aid before CI vs. 83% of the control group. Interestingly, these hearing aid users of the control group reported a significantly better quality of sound with their cochlear implant when compared to non-hearing aid users of the control group.

In this study, patients with MD had better pre-operative hearing (PTA4 = 103 dB HL) than patients with severe to profound hearing loss due to other pathologies (PTA4 = 122 dB HL), the difference being significant. This disparity has been reported in previous studies (19) and suggests that patients with MD, as a group, may undergo CI with relatively higher levels of residual hearing compared to that of the “general” population of cochlear implantees, since the residual hearing of patients with MD is not useful for communication even with a careful hearing aid fitting. Nevertheless, all patients who underwent CI in this study had bilateral severe to profound hearing loss (defined as PTA > 70 dB HL) with no benefit with hearing aids after a trial period, in accordance to the NICE guidance (20) and the American Academy of Otolaryngology—Head and Neck Surgery (10).

### Post-operative Hearing With the Cochlear Implant Is Similar in the MD and Control Groups

The hearing outcome of CI in patients with MD has been a matter of debate. McRackan et al. (21) evaluated 21 patients with MD and postulated that these patients achieve worse word recognition scores after CI than their standard sample of 178 adult implant recipients without MD, probably due to a nitric oxide-induced neuronal injury produced by endolymphatic hydrops. On the other hand, Chen et al. (22) suggested that extensive neuronal degeneration in the spiral ganglion is unusual in patients with MD, even in those undergoing a labyrinthectomy. Our post-operative speech discrimination results in quiet in patients with MD were similar to those of the control group, which supports the latter theory. In a similar way, Prenzler et al. (6) compared 27 implanted patients who have MD to a matched control group of cochlear implant users and concluded that speech understanding in the MD group was at least equal to that of “general population” CI recipients. Kocharyan et al. (23) also found no auditory differences between 24 patients with MD and an aged-matched control group of adults with cochlear implants.

### Post-operative QoL and Quality of Sound

Regarding QoL and quality of sound, the majority of patients from both groups reported a positive benefit following CI. This improvement was seen in the GBI with an overall benefit of +27 (MD group) and +29 (control group) after surgery. Similarly, there was a significant improvement in all the subdomains of the NCIQ (especially in “basic sound perception,” “speech production,” and “activity”) after CI and a moderate self-perception of auditory disability in the SSQ_12_. When the subjective quality of sound was studied, patients with MD and those in the control group reported a mean score of 79 and 70, respectively, which indicates a moderate benefit for both groups.

To the best of the authors' knowledge, this study represents the most extensive QoL research in MD patients who underwent CI (Table 5).

**Table 5:** QoL publications on patients with MD who undergo CI.

**References**	**Number of MD patients**	**Control group**	**Bilateral/unilateral MD**	**Ablative surgery on implanted ear**	**QoL test(s)**	**Results**	**Observations**
Kurz et al. (24)	8	No	4 Bilateral 4 Unilateral	No	MDOQ	QoL improvement (*p* = 0.035)	
Fife et al. (25)	10	No	Not specified	No	HHI MDFLS	Post-op HHI = 55.8 ± 25.3. Similar pre-op and post-op QoL according to MDFLS (*p* = 0.52).	No pre-op HHI.
Vermeire et al. (26)	7	No	Not specified	No	NCIQ	No pre-op evaluation. Poorer post-op NCIQ results than other studies.	
Perkins et al. (27)	3	No	Unilateral SSD	Labyrinthectomy	SSQ APHAB	Improvement in subjective QoL in all three patients.	No statistical analysis.
Mick et al. (28)	20	Yes*N* = 20	17 Bilateral 3 Unilateral	Not specified	SF36 (*N* = 12)	Improvement in functioning domain (*p* = 0.046) and trend to improvement in social functioning domain (*p* = 0.08).	Did not compare QoL to control group (only eight matched pairs completed QoL tests).
Canzi et al. (29)	4	No	4 Unilateral	Translabyrinthine neurectomy	MDPOSI	All parameters improved post-op.	No statistical analysis.
Present study	18	Yes*N* = 18	11 Bilateral 7 Unilateral	3 Previous vestibular neurectomy2 Simultaneous labyrinthectomy2 Sequential labyrinthectomy	NCIQ GBI SSQ_12_ HISQUI_19_	Post-op improvement with similar results to control group.	

*MD, Menière disease; QoL, quality of life; Pre-op. pre-operative; Post-op, post-operative; SSD, single side deafness; MDOQ: Menière disease outcome questionnaire; HHI, hearing handicap inventory; MDFLS, Menière disease-functional level scale; NCIQ, Nijmegen Cochlear Implant Questionnaire; SSQ, Speech, Spatial and Qualities of Hearing Scale; APHAB, abbreviated profile of hearing aid benefit; SF36, 36-item Short Form; MDPOSI, Menière disease patient oriented severity index; GBI, Glasgow Benefit Inventory; HISQUI, Hearing Implant Sound Quality Index*.

In most published studies, there is great variability in the QoL tests used and, more importantly, no specific hearing QoL tools were used. Vermeire et al. (26) assessed the QoL of seven unilateral CI patients with MD using the post-operative NCIQ. The authors reported mean values for the following subdomains: “basic sound perception” 46, “advanced sound perception” 34, “speech production” 64, “self-esteem” 51, “activity” 47, and “social interaction” of 46 points. All subdomain scores are lower than those found in the present study as well as in previous studies that used the NCIQ (13, 30).

The patients with MD in the present study showed similar health-related QoL than the control group with results similar to those reported in the literature (13, 30). However, in agreement with Vermeire et al., no correlation between speech perception and QoL was found (26). According to previous studies, the subdomain “advanced sound perception” seemed to be the most susceptible to the effect of CI, because it was correlated more often with the objective measures (13). As commonly stated in QoL studies, we think that other parameters such as the patient's capacity to perceive benefit could influence the correlation between the objective measures and some of the subdomains.

### Labyrinthectomy and CI

Surgical labyrinthectomy may be offered for patients with persistent MD who fail more conservative treatments. It can provide a definitive solution for vertigo attacks but this destructive technique involves the removal of the ipsilateral vestibular receptors and the cochlear function. The decompensation of the system can generate a sensation of dizziness and imbalance that it should be compensated by central, visual and proprioceptive mechanisms. But the final lost of residual hearing add an extra disability not always expected by patients and cochlear implantation becomes the only alternative.

Controversy also exists about performing simultaneous or sequential CI. On one hand, performing both surgeries at the same time reduces the duration of deafness, while avoiding the hypothetical risk of cochlea obliteration (9). On the other hand, although residual hearing in these patients is usually not useful, some prefer to delay CI until they are used to living with no residual hearing following the labyrinthectomy (31).

QoL parameters can be affect by audiological parameters and their own experience of vestibular function that it is difficult to quantify and it can be multifactorial.

The two patients who had simultaneous labyrinthectomy and CI in this study reported poor results in their quality of life and quality of sound. The patients were a 57-year-old man with unilateral MD and severe hearing loss following a radical cavity long time before, and a 70-year-old woman with bilateral MD. Both presented severe sensorineural hearing loss with contralateral anacusis. Following extensive and careful counseling, both decided to undergo simultaneous labyrinthectomy and CI due to frequent vertigo and drop attacks, as well as fluctuating hearing loss and aural symptoms on the ear to be implanted. Neither of the two patients reported any further attacks of vertigo after surgery, but they did report problems with their balance and dizziness attributed to poor compensation. After a two-year follow-up neither of the two patients had any more complaints about vestibular symptoms. Interestingly, the post-operative PTA4 and percentage of discrimination of disyllables in quiet of these particular patients were 33 dB and 75%, and 36 dB and 67%, respectively. They had better auditory results than both the whole MD group and the control group. In our opinion, this discrepancy between very good auditory results and the poor self-reported QoL scores may be explained by both the impact of bilateral vestibular hypofunction and the loss of residual hearing. In agreement with Hansen et al. (32), we believe that patients with pre-operative residual hearing and simultaneous labyrinthectomy and CI “will have not experienced the full consequences of deafness and may not fully appreciate the benefit of the cochlear implant for rehabilitation of the new deficit.”

On the other hand, two other patients had undergone labyrinthectomy 3 and 8 years before ipsilateral CI. As mentioned earlier, the possibility of cochlear obliteration must be considered following inner ear procedures. Both patients, a 68-year-old man with unilateral MD and a 65-year-old woman with bilateral MD, had good auditory and QoL results, similar to other cochlear implantees. Normal cochlear fluid signal was observed in the pre-operative MRI, and no difficulties were observed during electrode insertion. However, the patient with unilateral MD presented electrode extrusion 1 year after CI, but no difficulties were noted for reintroduction of the electrode array.

Limited evidence exists to date on cochlear obliteration after labyrinthectomy. Charlett and Biggs (31) reported that a third of patients who had undergone translabyrinthine removal of acoustic neuroma presented a partial or total obliteration of the cochlea in the MRI after 36 months of follow-up (range 4–185 months). The authors concluded that the time elapsed since the labyrinthectomy did not seem to be a predictor for obliteration. Nevertheless, Sargent et al. (33) conducted a study of 18 patients who had undergone transmastoid labyrinthectomy without internal auditory canal dissection. Results suggested that patency of the cochlea after surgery does not result in a loss of cochlear fluid signal in MRI, probably because there is no vascular compromise as in tumor removal. In agreement with these studies, Mukherjee et al. (8) found no MRI alterations or intraoperative difficulties in three patients undergoing CI and sequential labyrinthectomy, despite 2, 9, and 11 years of delay in surgery. In agreement with these results, Osborn et al. (34) reported that a woman who underwent CI, had good audiological outcomes and improved QoL 21 years after labyrinthectomy for MD treatment.

### Vestibular Neurectomy and CI

Three patients in the study presented here had undergone a previous retrosigmoid vestibular neurectomy on the ipsilateral implanted ear 15, 19, and 25 years earlier. All of them initially preserved their hearing thresholds, but a slow progressive decline in hearing thresholds led to severe to profound hearing loss. No MRI alterations were observed, and no complications were found during CI. These three patients had similar auditory results to the rest of the MD group, but they performed better in the subdomains “basic sound perception” (*p* = 0.038), “speech” (*p* = 0.005), “activity” (*p* = 0.038), and “social interactions” (*p* = 0.038) in the NCIQ.

To the best of our knowledge, there are no published studies that report on CI after vestibular neurectomy for MD. Nowadays, retrosigmoid vestibular neurectomy is less frequently performed as an alternative for refractory vertigo, even though the success rate is very high (89–96%) (35, 36). Hearing preservation (within 10 dB of the pre-operative level) can be achieved in the majority of patients (36, 37).

Even when the associated QoL results should be taken with caution due to the small sample size, our study suggests that CI can be a good solution for patients with MD who undergo vestibular neurectomy when hearing cannot be preserved during surgery, or if there is a post-operative decrease in hearing.

### Limitations

As most publications in this field, we report a retrospective study with a relatively small number of patients, which could limit the statistical significance of the results. Nevertheless, as shown in Table 5, to our knowledge, this is the only QoL study that includes QoL questionnaires that specifically focuses on cochlear implants and a matched control group.

Throughout the long follow-up of patients with MD, vestibular function was measured with different vestibular tests (including video head impulse test (vHIT), caloric and rotatory testing). However, due to the heterogeneity of tests conducted among patients, as well as the known lack of correlation of many of the results with the clinical findings, we have not included this information in this paper. In patients with MD, a personalized approach is recommended, and treatment decisions are mainly based on the clinical findings, especially the frequency of vertigo, drop attacks, and hearing impairment (4).

## Conclusion

This group of 18 patients with severe hearing loss and MD demonstrated excellent improvement in hearing and a significant QoL benefit after CI comparable to cochlear implant users with other conditions who were matched for demographic factors.

Delayed CI after transmastoid labyrinthectomy or retrosigmoid vestibular neurectomy can be performed and similar or better results can be expected as to those seen in other implanted patients. Delayed CI remains a viable treatment option when a normal cochlear fluid signal can be seen on T2-weighted MRI.

Patients undergoing simultaneous CI and labyrinthectomy may achieve similar hearing results as the population of cochlear implantees who did not require labyrinthectomy, but careful counseling is needed in this subset of patients.

## Data Availability Statement

The original contributions presented in the study are included in the article/supplementary material, further inquiries can be directed to the corresponding author/s.

## Ethics Statement

The studies involving human participants were reviewed and approved by CEICC La Paz University Hospital. The patients/participants provided their written informed consent to participate in this study.

## Author Contributions

IS-C, MC, JM-P, JG, TM, JP, MP, and LL provided substantial contributions to the conception or design of the work or the interpretation of data for the work, worked on the draft or revised it critically for important intellectual content, and agreed on accountability for all aspects of the work in ensuring that questions related to the accuracy or integrity of any part of the work are appropriately investigated and resolved. The final version was approved for publishing by all authors.

## Conflict of Interest

The authors declare that the research was conducted in the absence of any commercial or financial relationships that could be construed as a potential conflict of interest.
